# Using the new guideline for diagnosing anthelmintic resistance of gastrointestinal nematodes to different chemical components in sheep in the Rio Grande do Norte State, Brazil

**DOI:** 10.1590/S1984-29612025060

**Published:** 2025-10-24

**Authors:** Valdi de Lima, Jessica Caroline Nascimento Rodrigues, Francisca Fernanda da Silva, Victor Henneg Campelo de Lima, Viviany Lúcia Fernandes dos Santos, Juliete de Lima Gonçalves, Tamara Tais Tres, Carlo Aldrovandi Torreão Marques, Jacira Neves da Costa Torreão, Tairon Pannunzio Dias-Silva, Antonio Leandro Chaves Gurgel

**Affiliations:** 1 Universidade Federal do Rio Grande do Norte – UFRN, Departamento de Zootecnia, Natal, RN, Brasil; 2 Universidade Federal do Piauí – UFPI, Campus Professora Cinobelina Elvas, Bom Jesus, PI, Brasil

**Keywords:** Anthelmintic drugs, FEC, FECRT, gastrointestinal nematodes, Medicamentos anti-helmínticos, OPG, TRCOF, nematódeos gastrointestinais

## Abstract

The aim of this study was to diagnose anthelmintic resistance (AR) of gastrointestinal nematodes to different anthelmintics in crossbred Dorper X Santa Inês sheep using the new research guidelines of the World Association for the Advancement of Veterinary Parasitology. After estimating and identifying an approximate number of 10,000 sheep in the study area, a total of 256 sheep at different reproductive stages, presenting faecal egg count per gram of faeces (FEC) ≥ 400 were used. The faecal egg count reduction test (FECRT) was carried out to evaluate the pre- and post-treatment of anthelmintics, including albendazole, closantel, disophenol, ivermectin, levamisole, monepantel, moxidectin and trichlorfon, using the new classification criteria to analyze the results of an FECRT, which defined three possible classifications: susceptible, resistant and inconclusive. The highest average FEC was observed when the ewes were lactating, while the lowest FEC was observed in ewe lambs. Resistance was observed for albendazole, closantel, disophenol, ivermectin, levamisole and moxidectin. Two drugs (trichlorfon and monepantel) proved to be highly effective in treating gastrointestinal nematodes, presenting CI varying from 98 to 100% and 97 to 100%, respectively. Gastrointestinal nematodes from sheep raised in the in the Rio Grande do Norte State, Brazil are susceptible to the active principles trichlorfon and monepantel.

## Resumo

O objetivo deste estudo foi diagnosticar a resistência anti-helmíntica de nematódeos gastrintestinais a diferentes anti-helmínticos em ovinos mestiços Dorper X Santa Inês utilizando as novas diretrizes de pesquisa da Associação Mundial para o Avanço da Parasitologia Veterinária. Após estimar e identificar um número aproximado de 10.000 ovinos na área de estudo, foram utilizados um total de 256 ovinos em diferentes estágios reprodutivos, apresentando contagem de ovos fecais por grama de fezes (OPG) ≥ 400. O teste de redução da contagem de ovos fecais (TRCOF) foi realizado para avaliar o pré e pós-tratamento com anti-helmínticos, incluindo albendazol, closantel, disofenol, ivermectina, levamisol, monepantel, moxidectina e triclorfon, utilizando os novos critérios de classificação para analisar os resultados de um TRCOF, que definiu três possíveis classificações: suscetível, resistente e inconclusivo. A maior OPG média foi observada quando as ovelhas estavam em lactação, enquanto a menor OPG foi observada em cordeiras. Foi observada resistência a albendazol, closantel, disofenol, ivermectina, levamisol e moxidectina. Dois fármacos (triclorfom e monepantel) demonstraram alta eficácia no tratamento de nematoides gastrointestinais, apresentando IC variando de 98 a 100% e 97 a 100%, respectivamente. Nematódeos gastrointestinais de ovinos criados no Estado do Rio Grande do Norte, Brasil, são suscetíveis aos princípios ativos triclorfom e monepantel.

## Introduction

Sheep farming is a thriving activity in Brazil, contributing to the production of meat, wool, and leather products. Brazil has a herd totaling 18,410,551 sheep, with the majority being raised in the Northeast region (IBGE, 2015) with a unique characteristic: their meat is highly appreciated by the population and plays a crucial role in providing economic and nutritional support to many rural families (Adams et al., 2021; Nascimento et al., 2021). Despite the distinctive climatic challenges faced in the Northeast, including water scarcity and limited forage availability, the region demonstrates significant potential for productive sheep farming. This is primarily due to the adaptability and resilience of these animals to the environmental conditions.

Sheep farming in the Northeast is mainly focused on meat production and many technologies have been adopted to improve the results obtained by the animals (Bezerra et al., 2022). However, sheep suffer from diseases such as gastrointestinal nematode parasitosis, which is considered the biggest and most serious health problem for small ruminants (Chagas et al., 2022).

Managing gastrointestinal worms represents a significant challenge in advancing the sheep production sector, particularly in tropical regions where the economic repercussions are most pronounced (Vieira, 2008). The economic impacts caused by these infections are unmeasurable, but what it is known is that it includes decreased productivity and the unfortunate loss of many animals (Faria et al., 2016). Insufficient disease control measures can render the entire activity economically unsustainable.

The problem of this parasitosis has increased due, above all, to the adoption of the semi-intensive farming system (grazing animals with concentrated supplementation). Although this production method increases productivity, it also increases contamination of the environment, making it difficult to control the infection of the animals due to the increase in the stocking rate of the pasture (Moreira et al., 2011) and consequently the amount of gastrointestinal nematode eggs deposited in the pasture (Gazda et al., 2012). However, sanitary management has not been adapted to the new farming system and worm control is basically conducted using anti-parasitic drugs, but their use must follow the appropriate recommendations, otherwise the problem could worsen.

Anthelmintic drugs emerged in the 1960s as an alternative way of controlling the disease. At first, the method reduced losses, but the continuous and inadequate use of the compounds limited natural selection and encouraged pathogens to diversify into more resistant ones (Sargison et al., 2019; Santiago-Figueroa et al., 2019; Bassetto et al., 2024). As a result, parasites have acquired the so-called AR, which is when the dose of a drug no longer has the capacity to efficiently control the infection. In addition, parasites are showing multiple anthelmintic resistance (MAR) and this is worrying, given that there is already resistance to more than two chemical groups of drugs on the market (Bassetto et al., 2024).

Consequently, aside from the decline in productivity attributed to the disease, the presence of parasite resistance, if undetected, leads to even more substantial losses stemming from the inability to manage infections within the herd. As a result, the costs incurred also encompass investments in anthelmintic drugs, which, in turn, often fall short of expectations, along with the associated labor required for their administration (Vilela et al., 2018).

In order to overcome this common situation around the world, it is very important to evaluate the anthelmintic resistance of gastrointestinal nematodes. Thus, studying the effectiveness of anthelmintic drugs and diagnosing which drugs already have parasitic resistance can positively contribute to the development of more efficient strategies for controlling worm infections and thus increase the profitability of sheep farming. The FECRT remains the method of choice to assess drug efficacy, and hence the most commonly applied test for the diagnosis of AR (Kaplan & Vidyashankar, 2012).

Until a few years ago, the AR detection followed the methods and recommendations of the World Association for the Advancement of Veterinary Parasitology (WAAVP) (Coles et al., 1992). However, many new insights have been gained regarding the optimal experimental design for the FECRT (Torgerson et al., 2005; Levecke et al., 2012; Levecke et al., 2018). In view of this, the new guideline developed by Kaplan et al. (2023) addresses four important differences compared to the previous recommendations: to perform the FECRT based on pre- and post-treatment FEC of the same animals; requires a minimum total number of eggs to be counted under the microscope; flexibility in the size of the treatment group; and this new guideline address all major livestock species.

Thus, using the new guideline, the aim of this study was to assess the anthelmintic efficacy of different anthelmintics based on the faecal egg count reduction test (FECRT) in crossbred Dorper and Santa Inês ewes.

## Material and Methods

### Site of study and sheep farms

The experiment was conducted in different sheep farms in the state of Rio Grande do Norte, where according to Köppen and Geiger the climate classification is Aw, with an average temperature of 25.7°C, and average annual rainfall of 1107 mm. The study lasted from September 2015 to February 2016, totaling 14 collections. From a total of 10,000 sheep from seven farms, 256 animals were used to evaluate anthelminthic resistance. In each one of the seven flocks (on their own farm), the animals remained under normal farm management, on pasture cultivated with grasses of the genera *Urochloa* sp., *Panicum* sp. and *Digitaria* sp., managed under intermittent stocking.

Resistance was assessed through *in vivo* method (FECRT), as recently indicated by the World Association for the Advancement of Veterinary Parasitology guidelines (WAAVP) (Kaplan et al., 2023), with 90% confidence or credible intervals (CI).

To select the experimental animals, which were evaluated in the FECRT, the Famacha^©^ method was used to assess the coloration of the animals’ conjunctiva, where the animals’ degree of anemia is determined using an illustrative card. The card has five categories ranging from one (1) (bright red) to five (5) (pale, almost white), indicating different hematocrit values, from 1 to 5 respectively 35, 25, 20, 15 and 10% (Van Wyk & Bath, 2002). Therefore, based on the Famacha^©^ method, anemic animals were excluded, so as not to interfere with the animal’s response to the drug administered.

To conduct the experimental test, on each farm, a 10% sample of the animals was tested. The sample size was defined to be representative of the entire group of animals. From this sampling, only sheep presenting faecal egg count per gram of faeces (FEC) ≥ 400 were selected. Therefore, samples of 32 to 44 animals from 7 different sheep farms, totaling 256 females, at different physiological stages including ewe lambs, ewes in the breeding season, ewes in early and late pregnancy, and ewes in the breeding season where there was also weaning, as described in Table 1.

**Table 1:** Herd size within each sheep farm, general averages of faecal egg count (FEC) of ewes at different reproductive moments, before the anthelmintic administration in the different sheep farms studied.

**Sheep farm**	**Allocated animals (number)**	**Number of animals used in the effectiveness test (FEC ≥ 400)**	**FEC (D0°)**	**Reproductive Moment**
A	1470	32	2416.67^ab^	Early pregnancy
B	1984	36	1014.81^b^	Ewe lambs
C	885	32	2391.67^ab^	Late pregnancy
D	843	40	3400.00^a^	Lactation and breeding season
E	1075	36	1655.56^ab^	Late pregnancy
F	1300	36	1737.04^ab^	Middle of pregnancy
G	1000	44	3184.85^a^	Late pregnancy

Means followed by different lowercase letters in the column differ from each other (p < 0.05) according to the Tukey’s test.

Dietary management consisted of grazing during the day and returning to the facilities at night where they received protein supplementation and hay. Each sheep farm was evaluated for the effectiveness of three drugs and a control, respecting the minimum interval of 30 days from the last anthelmintic administration as recommended by Coles et al. (2006).

### Faecal sample collection

Faecal samples were taken directly from the rectal ampulla of each animal. using individual, identified plastic collection bags. These fecal samples were packaged in transparent plastic sealable bags individually and identified, placed in the styrofoam cooler with the chilled ice-packs, and carried to the Animal Parasitology Laboratory at the Jundiaí Agricultural School - Specialized Agricultural Sciences Unit of the Federal University of Rio Grande do Norte, where they were processed up to 48 hours after collection.

### Faecal egg counts

Quantitative analysis was conducted by faecal egg count per gram of faeces, according to the technique of Gordon & Whitlock (1939) modified by Ueno & Gonçalves (1998). Briefly, two grams of macerated faeces from each animal were used, which were homogenized in 58 mL of hyper-saturated sugar solution, strained and placed in a MacMaster chamber. After two minutes, eggs were counted using a compound light microscope. The total number of eggs per animal was determined by multiplying the number of eggs by 100.

### Faecal egg count reduction test

For the FECRT, the selected animals were treated with the anthelmintics that had previously been used on the property, in order to assess the effectiveness of the products. The drugs used for the anthelmintic resistance test are described in Table 2. The dosage and route of administration followed the recommendations of the manufacturers of each anthelmintic and according to animal body weight.

**Table 2:** Chemical groups of the drugs used, manufacturer, dose and route of administration.

**Chemical groups**	**Active principles**	**Manufacturer**		**Dose**	**route**
Imidatiazoles	Levamisole	Fort Dodge	1 mL/10 kg PV		Oral
Salicylanides	Closantel	Biogénesis Bagó	1 mL/10 kg PV		Oral
Organophosphates	Trichlorfon	Bayer	10 mL de sol. a 10%/10 kg PV		Oral
Benzimidazoles	Albendazole	Hipra	0.35 mL/10 Kg PV		Oral
Macrocyclic lactones	Ivermectin	Tortuga	0.2 mL/10 kg PV		Subcutaneous
	Moxidectin	Zoetis	(0.05 mL/kg)		Subcutaneous
Nitrophenol substitutes	Disophenol	Ibasa	0.5 mL/10 kg PV		Subcutaneous
Aminoacetonitrile derivatives	Monepantel	Novartis	1 mL/10 kg PV		Oral

The animals were evenly distributed in the experimental groups and each group contained the same number of animals. The groups were defined as the treated groups (T) and the control group (C). The control group was included to observe any natural decline or increase in the FEC as suggested by Neves et al. (2014).

The day (D0) of administration was considered FEC_1_ and fifteen days (D15) post-treatment was considered FEC_2_, in which a new faecal sample was collected and used for individual FEC. The values found in the FEC_1_ and FEC_2_ were used to determine FECRT with upper and lower bounds of the 90% confidence (credible) intervals.

### Data analysis

The faecal egg count reduction (FECR) was estimated using the SHINY tool, a web interface developed by the ‘shiny-egg-Counts’ project, which provides an intuitive web interface for analysing FEC data (Kaplan et al., 2023). This tool calculates the efficacy based on FEC pre- and post-treatment within the same group, allowing the analysis of paired samples, according to WAAVP (Kaplan et al., 2023). Also, this tool deals with the zero-inflation component captures the excess number of zero counts that could come from unexposed animals and it use a hierarchical model that uses binomial distribution to capture the counting variability, and a Poisson-gamma distribution to model the overdispersion (Wang et al., 2017).

Kaplan et al. (2023), using the new classification criteria for analyzing the results of an FECRT outlined by Denwood et al. (2023), defined three possible classifications:

1 - Susceptible: when the lower 90% CI is greater than or equal to the lower efficacy threshold (corresponding to the lower limit of the grey zone) and the upper 90% CI is greater than or equal to the expected efficacy (corresponding to the upper limit of the grey zone).
2 - Resistant: when the upper 90% CI is less than the expected efficacy (corresponding to the upper limit of the grey zone). This includes the sub-classification of ‘low resistant’, which meets the additional criteria of the lower 90% CI being greater than or equal to the lower efficacy threshold (lower limit of the grey zone).
3 - Inconclusive: if neither of these criteria are met.

Reproductive Moments data were subjected to analysis of variance and the means were compared by the Tukey’s test at a 5% significance level.

## Results

Before the anthelmintic were administered, the highest FEC averages were found in ewes at the end of pregnancy (sheep farm G) and in lactation (sheep farm D). The FEC count in ewe lambs (sheep farm B) differed (p < 0.05) from the other categories studied (Table 1).

Faecal egg count of pre (FEC_1_) and post-treatment (FEC_2_), followed by the results of FECRT, CI and test outcome according to the anthelmintic used are shown in Table 3, for each sheep farm studied. In general, according to CI there was a greater effectiveness when using monepantel (Aminoacetonitrile derivatives) (97 - 100%) and trichlorfon (Organophosphates) (98 - 100%) and, FECR of 100 and 99%, respectively, indicating high sensitivity to these active principles. It should also be noted that ivermectin (Macrocyclic lactones) had no effect against gastrointestinal nematodes in the farms studied.

**Table 3:** Mean faecal egg counts ± standard deviation (FEC ± SD) of pre- (FEC_1_) and post-treatment (FEC_2_), faecal egg count reduction test (FECRT) with anthelmintic efficacy percentage, lower and upper 90% credible intervals (UI) and test outcome from SHINY at each sheep farm studied.

**Sheep farm A**					
**Groups**	**FEC_1_**	**FEC_2_**	**FECRT (%)**	**Credible intervals (%)**	**Test outcome**
Albendazole	2338 ± 2488	1088 ± 896	53	41 - 64	**Resistant**
Moxidectin	1850 ± 2373	850 ± 652	54	39 - 65	**Resistant**
Ivermectin	3063 ± 1697	3063 ± 2977	2	0 - 15	**Resistant**
Control	2138 ± 3644	1363 ± 988	37	19 - 49	
**Sheep farm B**					
Groups	FEC_1_	FEC_2_	FECRT (%)	Credible intervals (%)	Test outcome
Albendazole	1022 ± 1102	5722 ± 10762	0	0 - 1	**Resistant**
Moxidectin	1178 ± 713	5156 ± 3168	0	0 - 2	**Resistant**
Ivermectin	844 ± 1223	5878 ± 3075	0	0 - 1	**Resistant**
Control	1944 ± 2355	4244 ± 3017	0	0 - 3	
**Sheep farm C**					
Groups	FEC_1_	FEC_2_	FECRT (%)	Credible intervals (%)	Test outcome
Moxidectin	2650 ± 2073	1663 ± 947	36	22 - 49	**Resistant**
Closantel	2200 ± 1684	925 ± 868	58	44 - 68	**Resistant**
Ivermectin	2325 ± 2518	4388 ± 7167	0	0 - 3	**Resistant**
Control	1525 ± 1754	1550 ± 1016	0	0 - 2	
**Sheep farm D**					
Groups	FEC_1_	FEC_2_	FECRT (%)	Credible intervals (%)	Test outcome
Albendazole	2600 ± 1833	1200 ± 1572	52	41 - 62	**Resistant**
Moxidectin	3660 ± 1584	2620 ± 1316	31	2 – 42	**Resistant**
Closantel	3860 ± 3625	1280 ± 2533	31	2 - 42	**Resistant**
Control	5260 ± 5788	2020 ± 1720	61	54 - 67	
**Sheep farm E**					
Groups	FEC_1_	FEC_2_	FECRT (%)	Credible intervals (%)	Test outcome
Disophenol	1900 ± 2027	656 ± 887	65	54 - 75	**Resistant**
Levamisole hydrochloride	1722 ± 1981	900 ± 775	61	49 - 72	**Resistant**
Monepantel	1344 ± 881	0 ± 0	100	97 - 100	Susceptible
Control	2411 ± 1127	1611 ± 1886	33	16 - 45	
**Sheep farm F**					
Groups	FEC_1_	FEC_2_	FECRT (%)	Credible intervals (%)	Test outcome
Albendazole	1500 ± 1035	3298 ± 4045	0	0 - 4	**Resistant**
Disophenol	1700 ± 1526	678 ± 373	60	47 – 71	**Resistant**
Trichlorfon	2011 ± 2235	333 ± 563	83	76 - 89	**Resistant**
Control	1222 ± 1036	11011 ± 6729	0	0 - 1	
**Sheep farm G**					
Groups	FEC_1_	FEC_2_	FECRT (%)	Credible intervals (%)	Test outcome
Albendazole	5173 ± 6764	3500 ± 4601	32	22 - 41	**Resistant**
Disophenol	1818 ± 1286	2582 ± 4766	0	0 - 6	**Resistant**
Trichlorfon	2564 ± 2219	18 ± 40	99	98 - 100	Susceptible
Control	2427 ± 2370	4973 ± 9049	0	0 - 2	

On sheep farm A was identified resistance to Albendazole (41 – 64% CI), Moxidectin (39 - 65% CI) and Ivermectin (0 - 15% CI). On sheep farm B was identified resistance to Albendazole (0 – 1% CI), Moxidectin (0 - 2% CI) and Ivermectin (0-1% CI). On sheep farm C was identified resistance to Moxidectin 22 – 49% CI), Closantel (44 - 68% CI) and Ivermectin (0 - 3% CI). On sheep farm D was identified resistance to Albendazole (41 – 62% CI), Moxidectin (2 - 42% CI) and Closantel (2 - 42% CI). On sheep farm E was identified resistance to Disophenol (54 – 75% CI), Levamisole hydrochloride (49 - 72% CI) and susceptibility to Monepantel (97 - 100% CI). On sheep farm F was identified resistance to Albendazole (0 – 4% CI), Disophenol (47 - 71% CI) and Trichlorfon (76 - 89% CI). On sheep farm G was identified resistance to Albendazole (22 – 41% CI), Disophenol (0 - 6% CI) and susceptibility to Trichlorfon (98 - 100% CI).

## Discussion

The initial diagnosis showed that ewe lambs had lower FEC, probably due to lower nutritional requirements and the reproductive stage they were in, with a consequent better immune capacity to respond to gastrointestinal parasites. The increase in FEC seen at the end of pregnancy and the beginning of lactation was characteristic of the so-called peripartum phenomenon (Amarante et al., 1992; Pereira et al., 2020). During this phase, ewes are more susceptible and there is an increase in the number of eggs in the faeces due to the greater fecundity of the adult worms, which only reduces after weaning, with the re-establishment of the effective immune response.

Based on CI of the FECRT, the gastrointestinal nematodes proved to be totally resistant to ivermectin, regardless of their physiological stage, which was possibly due to the prolonged use of the product (Torres-Acosta et al., 2019). For many years, the ineffectiveness of ivermectin has been reported for the treatment of sheep populations around the world (Ahid et al., 2008; Pereira et al., 2008; Byaruhanga & Okwee-Acai, 2013; Mahieu et al., 2014).

In general, a variation in the effectiveness of anthelmintics was verified between the different sheep farms. This evidence shows a very worrying situation, since six of the eight anthelmintics tested were ineffective in controlling gastrointestinal nematodes. The ineffective anthelminthics were ivermectin, albendazole, closantel, levamisole, disophenol and moxidectin. In these situations of drug resistance. It is noteworthy that our results demonstrated this condition (resistance and susceptibility) between the years 2015/2016. The diagnosis of anti-helminthic resistance to the various active ingredients available on the market must be constantly evaluated, always taking into account new evaluation techniques, seeking to identify products that are still effective and the implementation of new methods of controlling internal parasites, whether the combination of active ingredients (Bichuette et al., 2015; Coelho et al., 2015), sustainable worm control strategies such as pasture management (Quadros & Burke, 2024), dietary supplementation (Quadros & Burke, 2024), and targeted selective treatments (Marques et al., 2018). Therefore, measures to extend the shelf life of currently available anthelmintics need to be implemented urgently.

It was observed wide variation in egg counts per gram of feces between farms, both pre- and post-treatment. It is noteworthy that environmental, immunological, and management factors directly influence this variability, as there are different levels of susceptibility between animal categories, which is also influenced by the farm's nutritional and health management characteristics (Marques et al., 2018).

On sheep farm A, the upper and lower bounds of the 90% CI of the albendazole and moxidectin treatments were very close. However, the values recorded low percentages of effectiveness, classifying them as insufficiently active, indicating high resistance, showing that, on this sheep farm, gastrointestinal parasites presented a high degree of resistance to the active principles used. More serious than these chemical groups mentioned, we verified that, ivermectin did not have the slightest effect.

Even though in sheep farm B the females have better immune response (ewe lambs, under 12 months old), extreme anthelminthic resistance was found to albendazole, moxidectin and ivermectin, therefore, given the ineffectiveness of the products, there was a large increase in FEC_2_. On sheep farm C, closantel led to a reduction in FEC_2_, differing from the other anthelmintic drugs, but none of the principles had a satisfactory result.

On sheep farm D, despite the reduction on the FEC all the chemical groups used were insufficiently active. This reduction could also be the result of the peripartum phenomenon which can develop larvae in hypobiosis (inhibited larval development), linked to the fact that the lambs are being separated from the ewes and they are acquiring a better immune response.

The use of monepantel on sheep farm E was highly effective, showing high susceptibility of gastrointestinal nematodes to this active principle, being the only active principle capable of reducing the count to levels below 400 FEC, an indication of a mild parasitic infection or absence of parasites. This is possibly due to the fact that the product was recently launched (launched on the world market in 2010 and in Brazil in 2012) on the market for worm control and the animals had not had contact with this anthelmintic previously. However, there have already been reports of anthelmintic resistance to this chemical compound (Cintra et al., 2016; Mallmann et al., 2018; Bartley et al., 2019; Turnbull et al., 2019; Niciura et al., 2020; Viana et al., 2021).

According to Oliveira et al. (2017), resistance to monepantel occurred mainly in the farms that did not take any action aiming at delaying the resistance. It should be emphasized that the present experiment was conducted between 2015 and 2016. Based on this and according to research (Scott et al., 2013; Mederos et al., 2014; Van den Brom et al., 2015; Cintra et al., 2016), the efficacy of monepantel, which was found in the present research, should be recurrently evaluated, under similar climate and management conditions as well as under different conditions.

Trichlorfon-based deworming on sheep farms F and G proved to be efficient (< 400 FEC per gram). However, based on classification criteria established in 2023 by WAAVP, was effective only on farm G. The effectiveness of the active ingredient trichlorfon has been corroborated by other authors (Falbo et al., 2009; Oliveira et al., 2014), who report that the lower frequency of use of this drug contributes to a more efficient control of endoparasites, due to its lack of practicality at the time of administration, given that the form of administration (oral) and its presentation (in powder form) require prior dilution, as well as the high toxicity of the organophosphate group.

The occurrence of anthelmintic resistance to albendazole, closantel, disophenol, ivermectin, levamisole and moxidectin occurs due to multiple repetitions of use, indiscriminate use (Veríssimo et al., 2012; Bassetto et al., 2024**)**, without technical prescription and underdosages. The data presented proves the occurrence of MAR in all the sheep farms evaluated, since the parasites showed to be resistant to more than two pharmacological bases (Fissiha & Kinde, 2021). Reports of anthelmintic resistance to multiple drugs in individual parasite species, and in multiple parasite species across virtually all livestock hosts, are increasingly common (Kaplan & Vidyashankar, 2012; McMahon et al., 2013; Batista et al., 2023; Borges et al., 2024). The results obtained in this experiment showed that only 25% of medications were effective in controlling gastrointestinal helminths, that is, 2 drugs presented satisfactory results based on classification criteria with a confidence interval greater than ≥ 97%, depending on the sheep farm.

It can be concluded that gastrointestinal nematodes were susceptible to the active principles monepantel and trichlorfon, which presented high efficiency in controlling gastrointestinal nematodes in sheep from the Rio Grande do Norte State, Brazil.

In this scenario, sustainable worm control strategies, such as information about epidemiology, sheep management practices (especially better nutrition), targeted selective treatments and Selection of genetically superior animals for the characteristic of resistance to infections, which can delay the establishment of resistance to new and future anthelmintics need to be implemented urgently.

## Data Availability

The data that support the findings of this study are included in the manuscript but are available from the corresponding author on reasonable request.
